# Sex difference in circulating soluble form of ACE2 protein in moderate and severe COVID-19 and healthy controls

**DOI:** 10.3389/fmed.2022.1058120

**Published:** 2022-12-08

**Authors:** Josefina Robertson, Bengt Nellgård, Lillemor Mattsson Hultén, Staffan Nilsson, Keti Dalla, Mats Börjesson, Henrik Zetterberg, Joar Svanvik, Magnus Gisslén

**Affiliations:** ^1^Department of Infectious Diseases, Institute of Biomedicine, Sahlgrenska Academy, University of Gothenburg, Gothenburg, Sweden; ^2^Region Västra Götaland, Sahlgrenska University Hospital, Gothenburg, Sweden; ^3^Department of Anaesthesiology and Intensive care, Institute of Clinical Sciences, Sahlgrenska Academy, University of Gothenburg, Gothenburg, Sweden; ^4^Department of Molecular and Clinical Medicine, Institute of Medicine, Sahlgrenska Academy, University of Gothenburg, Gothenburg, Sweden; ^5^Department of Laboratory Medicine, Institute of Biomedicine, Sahlgrenska Academy, University of Gothenburg, Gothenburg, Sweden; ^6^Center for Health and Performance, Institute of Medicine, Sahlgrenska Academy, University of Gothenburg, Gothenburg, Sweden; ^7^Department of Psychiatry and Neurochemistry, Institute of Neuroscience and Physiology, Sahlgrenska Academy, University of Gothenburg, Gothenburg, Sweden; ^8^Clinical Neurochemistry Laboratory, Sahlgrenska University Hospital, Gothenburg, Sweden; ^9^Department of Neurodegenerative Disease, University College London (UCL) Institute of Neurology, London, United Kingdom; ^10^United Kingdom Dementia Research Institute, University College London (UCL), London, United Kingdom; ^11^Hong Kong Center for Neurodegenerative Diseases, Clear Water Bay, Kowloon, Hong Kong SAR, China; ^12^The Transplant Institute, Sahlgrenska University Hospital, Gothenburg, Sweden

**Keywords:** COVID-19, disease severity, sex, intensive care unit, sex difference, angiotensin-converting enzyme 2 (ACE2)

## Abstract

**Introduction:**

Membrane-bound angiotensin-converting enzyme-2 (ACE2) in epithelial cells is the main receptor for SARS-CoV-2. The extracellular portion of ACE2 may be shedded to plasma in which process ADAM17 (a disintegrin and metalloproteinase 17) is important. Results on the relationship between circulating levels of the soluble form of ACE2 (sACE2) and disease severity are inconclusive. This study investigates if sACE2 concentration correlates with COVID-19 severity, and whether this is affected by sex.

**Materials and methods:**

Soluble form of ACE2 was analyzed in three groups: 104 patients (23 women and 81 men) with severe COVID-19 admitted to an intensive care unit (ICU), patients with moderate COVID-19 who required hospital care (*n* = 19, 4 women and 15 men), and age and sex matched healthy controls (*n* = 20, 4 women and 16 men). Blood samples were collected at hospital admission between 18 March 2020, and 3 May 2021, and at follow-up between 27 October 2020, and 19 October 2021. Circulating sACE2 (μg/L) was measured in EDTA plasma with a sensitive enzyme-linked immunosorbent assay. Additionally, CRP, ferritin, and lymphocyte count were analyzed during hospital stay.

**Results:**

In total, 23 patients (22%) died in the ICU. When comparing healthy controls [mean age 58.1 (SD 11.4) years] and patients with moderate COVID-19 [mean age 61.0 (SD 13.2) years] with patients in the ICU [mean age 63.6 (SD 11.6) years], we found that sACE2 concentration decreased (70% reduction) with disease severity in men (*p* = 0.002) but increased 3.7-fold with severity in women (*p* = 0.043), suggesting a sex-related difference in how COVID-19 severity is related to sACE2 concentration. Moreover, we identified a relationship between inflammatory biomarkers and sACE2 concentration during the intensive care treatment, such that higher CRP and higher ferritin concentration correlated with lower sACE2 concentration in men.

**Conclusion:**

The decrease in sACE2 concentration, selectively in men, in severe COVID-19 is of pathophysiological interest since men are affected more severely by the disease compared to women. Additionally, the inflammatory biomarkers, CRP and ferritin, correlated inversely with sACE2 concentration, suggesting a role in severe disease. Our findings imply that sACE2 is a possible biomarker of disease severity in a sex-specific manner.

## Introduction

Membrane-bound angiotensin-converting enzyme 2 (ACE2) in epithelial cells is the main receptor for the severe acute respiratory syndrome coronavirus 2 (SARS-CoV-2). The Transmembrane Serine Protease 2 (TMPRSS2) also plays an important role (1). Another enzyme, a disintegrin and metalloproteinase 17 (ADAM17), may cause shedding of ACE2 from cells in the epithelia and from exosomes. This is supposed to have an important regulatory function in the immune system (2, 3).

It is known since long that angiotensin II differently affects the regional blood circulation in various tissues (4). The enzymatic activity of ACE2 is to convert angiotensin II to angiotensin 1–7, attenuating the effects of angiotensin II including vasoconstriction and inflammation (5, 6). Circulating soluble ACE2 (sACE2) may depend on the density of membrane-bound ACE2 in epithelial cells but also on the local ADAM17 activity. Thus, sACE2 may not entirely reflect the expression of ACE2 on epithelial cells. ACE2 expression varies in different tissues and the density in the small intestine seems to be high, thereby representing a major source of the enzyme entering the circulation (6). The balance between sACE2 and the tissue levels is not determined so far. It has been suggested that circulating sACE2 may protect from tissue infection by trapping SARS-CoV-2, and therapeutic attempts are even made to engineer human sACE2 to optimize binding to the spike protein in the virus (7). sACE2 may be analyzed with methods to evaluate the specific protein content as proteomics like OLINK (8–10), mass spectrometry (11), and enzyme-linked immunosorbent assay (ELISA) (12, 13), as well as, with enzymatic methods (14–18).

Early in the coronavirus disease 2019 (COVID-19) pandemic it was suggested in observational studies that the renin-angiotensin-aldosterone system (RAAS)-blockade by ACE inhibitors or angiotensin II type-I receptor blockers (ARBs) would increase the risk of severe SARS-CoV-2 outcomes by upregulating the expression of membrane-bound ACE2. However, many of these studies included a critical risk of confounding or selection bias, and the initial finding that RAAS inhibitor use increases the risk of severe COVID-19 has not been confirmed in later high-quality studies (19).

There is a strong support for a role of ACE2 and TMPRSS2 in severe COVID-19, and sACE2 has been proposed as a potential predictor of disease severity (20–22). Several reports have also tried to investigate the relationship between sACE2 and disease severity. However, studies of circulating sACE2 in severe COVID-19 have shown confusing results, such that plasma levels of sACE2 may be raised or reduced. Differences in enzymatic, ELISA, and immunoprecipitation methods make it difficult to compare the results, which may explain the divergence of sACE2 in different COVID-19 studies. Further, the population samples studied are often heterogenous with regard to age and gender. It is well-known that sACE2 in healthy men are higher than in healthy women (23). Despite this, the sACE2 response to moderate and severe COVID-19 has not been studied separately in men and women although being of possible importance in view of the higher probability of severe COVID-19 progression in men. Moreover, most previous studies have been cross-sectional without longitudinal follow-up of changes. In summary, the results on the relationship between levels of sACE2 and severity of COVID-19 are controversial and not entirely conclusive, and analyzes regarding sex differences are lacking.

The aim of the present study was to investigate sACE2 concentration in relation to COVID-19 severity, and potential associations with sex.

## Materials and methods

### Participants

In total, 104 patients with COVID-19, who were admitted to an intensive care unit (ICU) at the Sahlgrenska University Hospital, Gothenburg, Sweden, were included in the study (severe/critical COVID-19). All of them received mechanical ventilation. For comparison analyses, we included 19 patients with moderate COVID-19 who required hospital care but were not high flow nasal oxygen (HFNO)-dependent, at the Department of Infectious Diseases at the Sahlgrenska University Hospital, Gothenburg, Sweden (24). All cases were confirmed with reverse transcriptase polymerase chain reaction (RT-PCR) from nasopharyngeal and throat aspirates. Additionally, twenty healthy age and sex matched volunteers, mostly health care workers, were included as controls. The study was a sub-study of an ongoing prospective COVID-19 cohort study (25, 26), and was conducted in accordance with the ethical principles set out in the declaration of Helsinki. It was approved by the Swedish Ethical Review Authority (Dnr: 2020-01771). Written informed consent was obtained from all participants.

### Blood sampling and laboratory analyses

Blood samples at hospital admission were collected between 18 March 2020, and 3 May 2021. Blood samples at follow-up were collected between 27 October 2020, and 19 October 2021. Concentration of sACE2 (μg/L) was measured in EDTA plasma with an enzyme-linked immunosorbent assay (ELISA) using the High Sensitivity Human Soluble Angiotensin-Converting Enzyme 2 (ACE2) immunoassay (Catalog No. SK00707-06, Aviscera Bioscience Inc., Santa Clara, CA) according to the manufacturer’s instructions. The inter-assay coefficient of variation was <10%. Samples with sACE2 concentrations below the lower limit of detection (<0.3 μg/L, *n* = 8) were adjusted to 0.15 μg/L, and samples with sACE2 concentrations above the upper limit of detection (>631 μg/L, *n* = 2) were excluded due to uncertain values related to the possible impact of heterophilic antibodies. Concentration of C-reactive protein (CRP) and ferritin were analyzed using standard laboratory techniques and automated Alinity Instruments (Abbott Laboratories, Chicago, IL, USA). Lymphocyte count was measured in whole blood with the auto-hematology analyzer ADVIA^®^ 2120i System (Siemens Healthcare GmbH, Erlangen, Germany). All analyses were performed at the Department of Clinical Chemistry, Sahlgrenska University Hospital, Gothenburg, Sweden.

### Statistics

Descriptive statistics are shown for all variables involved in the analyses, presented as means with standard deviations and medians with interquartile ranges. For statistical analyses, continuous variables were log_10_ transformed. Student’s *t*-test was used for group comparisons and stratified by sex or severity. Associations between numeric variables were analyzed with linear regression and measured with Pearson correlation. Some associations were stratified by sex, and slope differences were investigated by an interaction term.

All statistical analyses were performed with the Statistical Package for the Social Sciences (SPSS) software version 25 (SPSS, Chicago, Illinois, USA) or Prism (GraphPad software version 8.0, La Jolla, California, USA). A significance level of 0.05 was used.

## Results

Of the 104 patients aged 23–85 years [mean age 63.6 (SD 11.6) years] with COVID-19 requiring intensive care treatment, including mechanical ventilation, 23 (22%) were women. A total of 23 patients (22%) died in the ICU. Blood samples were collected at hospital admission [mean 11.5 (SD 6.4) days since symptom onset] and at follow-up [mean 227.5 (SD 39.8] days since symptom onset) (Table 1). Moreover, we identified 19 patients aged 37–79 years [mean age 61.0 (SD 13.2) years] with moderate COVID-19, not requiring ICU treatment but who were admitted to hospital. Four of these patients (21%) were women. None of the patients in this group died during follow-up. Blood sampling at hospital admission was performed in average 13.5 (SD 7.7) days since symptom onset, and at follow-up, in average, 241.5 (SD 48.3) days since symptom onset. The healthy controls were aged 42–81 years [mean age 58.1 (SD 11.4)], and four of them (20%) were women (Table 1).

**TABLE 1:** Descriptive statistics of included COVID-19 patients and healthy controls.

	COVID-19 in ICU* *n* = 104	Moderate COVID-19 *n* = 19	Healthy controls *n* = 20
Age, mean (SD)	63.6 (11.6)	61.0 (13.2)	58.1 (11.4)
Women, n (%)	23 (22)	4 (21)	4 (20)
Died, n (%)	23 (22)	–	–
Hypertension, n (%)	46 (44)	9 (47)	1 (5)
Diabetes mellitus, n (%)	24 (23)	5 (26)	1 (5)
Obesity, n (%)	26 (25)	6 (32)	**
Chronic heart disease***, n (%)	14 (13)	1 (5)	–
Corticosteroid treatment, n (%)	67 (64)	6 (32)	–
Tocilizumab (RoActemra) treatment, n (%)	2 (2)	–	–
Blood sampling at hospital admission, n (%)	77 (74)	19 (100)	19 (95)
Days since symptom onset, mean (SD)	11.5 (6.4)	13.5 (7.7)	–
sACE2 (μg/L), mean (SD)	30.5 (55.1)	58.9 (109.3)	42.7 (52.8)
sACE2 (μg/L), median (IQR)	9.4 (3.4–28.5)	12.0 (6.7–54.0)	21.0 (6.3–50.3)
Blood sampling at follow-up, n (%)	80 (77)	19 (100)	–
Days since symptom onset, mean (SD)	227.5 (39.8)	241.5 (48.3)	–
sACE2 (μg/L), mean (SD)	44.0 (84.2)	65.1 (122.7)	–
sACE2 (μg/L), median (IQR)	14.0 (5.1–38.5)	15.0 (4.7–37.0)	–

*Critical disease. **There was no information regarding BMI in healthy controls. ***Includes coronary heart disease, heart failure, cardiomyopathy. ICU, intensive care unit; SD, standard deviation; sACE2, soluble angiotensin-converting enzyme 2; IQR, interquartile range.

**FIGURE 1:**
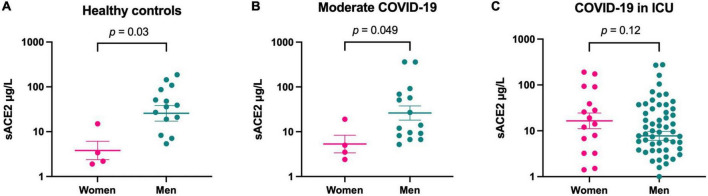
Soluble form of ACE2 (sACE2) concentrations (μg/L) in healthy controls **(A)**, at hospital admission for patients with moderate COVID-19 **(B)**, and at hospital admission for patients with COVID-19 in the intensive care unit (ICU) **(C)**, divided by sex. Individual values and mean value with SEM are shown for each group. Student’s *t*-test was used for group comparisons.

When comparing sACE2 concentrations between men and women in each severity group, we found that women displayed significantly lower levels among healthy controls, as expected, but also among patients with moderate COVID-19. However, there was no difference between men and women among patients with severe COVID-19 treated in the ICU (Figure 1). sACE2 concentrations did not differ between patients who died in the ICU and survivors (data not shown), and sACE2 levels did not correlate to age or days since symptom onset in any of the COVID-19 severity groups (Supplementary Figures 1, 2).

**FIGURE 2:**
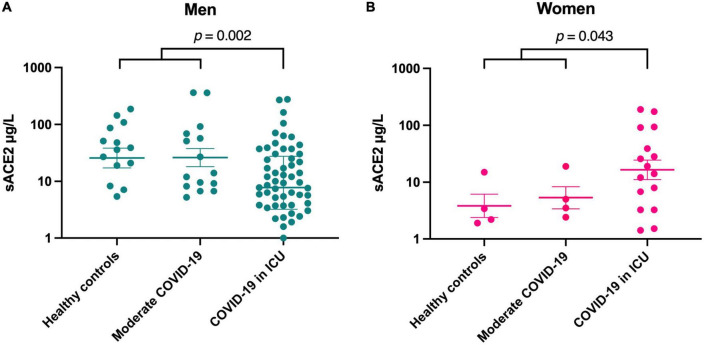
Soluble form of ACE2 (sACE2) concentrations (μg/L) in men **(A)** and women **(B)**. Individual values and mean value with SEM are shown for each group. Student’s *t*-test was used for group comparisons (ICU, intensive care unit).

Healthy controls and patients with moderate disease displayed similar sACE2 concentrations without any significant difference, regardless of sex (Figure 2). Therefore, we merged these groups and compared them as one group to patients with severe disease. In these analyses, severely ill men treated at an ICU displayed lower levels of sACE2 (70% reduction, *p* = 0.002) than men with moderate COVID-19 and healthy controls (Figure 2). Conversely, women with severe disease had significantly higher sACE2 values (3.7-fold increase, *p* = 0.043) than the group of women with moderate disease and healthy controls (Figure 2). Thus, our data suggest that sACE2 concentrations decrease with the severity of COVID-19 among men whereas contrariwise sACE2 concentrations increase with disease severity among women.

**FIGURE 3:**
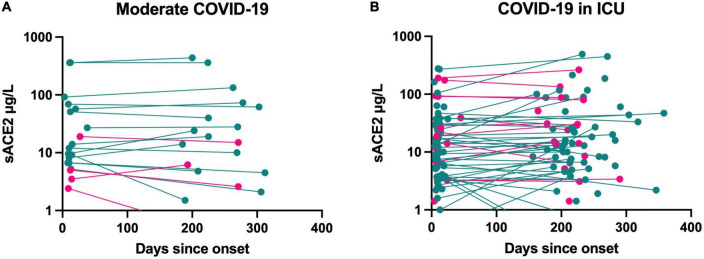
Longitudinal measurements of sACE2 (μg/L) in patients with moderate COVID-19 (*n* = 19) **(A)** and with COVID-19 requiring intensive care (*n* = 104) **(B)**. Men = green, women = pink (ICU, intensive care unit).

**FIGURE 4:**
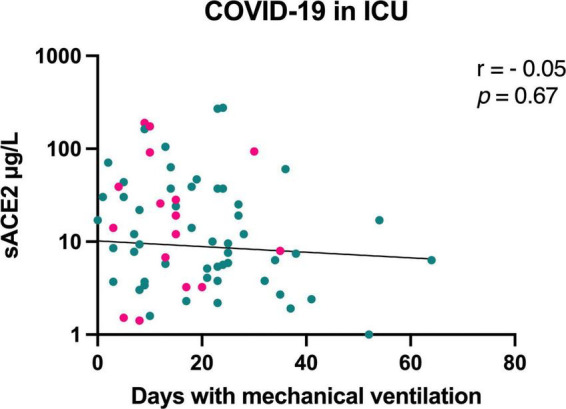
Correlation between sACE2 level (μg/L) at hospital admission and days with mechanical ventilation for patients with COVID-19 in the intensive care unit (ICU). Men = green, women = pink.

In a longitudinal analysis of sACE2 levels at hospital admission and at follow-up, we found no change in sACE2 over time in moderately ill or severely ill COVID-19 patients (Figure 3). Moreover, six patients with moderate COVID-19 and 67 patients with severe COVID-19 received corticosteroid treatment. The sACE2 levels, however, did not differ at hospital admission or follow-up between patients with or without corticosteroid treatment (Supplementary Figure 3). Only two COVID-19 patients in the study were treated with the IL-6 receptor blocking antibody tocilizumab (RoActemra), and its effect on sACE2 levels could therefore not be studied. When analyzing preexisting treatment with ACE inhibitors or ARBs among patients with moderate COVID-19 (*n* = 9) and COVID-19 in the ICU (*n* = 34), there was no significant difference in sACE2 levels in either group (Supplementary Figure 4). Among patients treated in the ICU, sACE2 concentration did not correlate with days with mechanical ventilation (Figure 4). Nor was there any correlation if only including men in this analysis (*r* = −0.03, *p* = 0.83).

To investigate if cardiovascular comorbidities affected the sACE2 concentration among patients in the ICU, we compared patients with and without hypertension (*n* = 44), diabetes mellitus (*n* = 20) and chronic heart disease (coronary heart disease, heart failure, cardiomyopathy; *n* = 14). There were no significant differences in sACE2 levels for any of these comorbidities (Supplementary Figure 5). Further, sACE2 concentration did not correlate with BMI (*r* = −0.07, *p* = 0.58).

**FIGURE 5:**
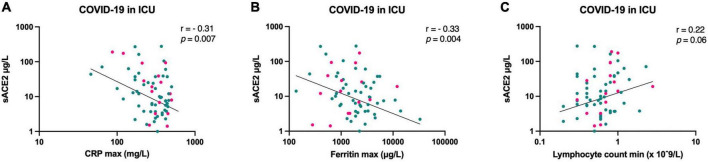
Correlation between sACE2 concentrations (μg/L) at hospital admission and maximum C-reactive protein (CRP, mg/L) **(A)**, maximum ferritin (μg/L) **(B)**, and minimum lymphocyte count (x 10^9^/L) **(C)**, respectively, for patients with COVID-19 in the intensive care unit (ICU). Men = green, women = pink.

The relationship between inflammatory biomarkers and sACE2 levels during the intensive care was studied in correlation analyses including maximum levels of C-reactive protein (CRP, mg/L), maximum concentrations of ferritin (μg/L), and minimum lymphocyte counts (×10^9^/L), recorded during hospitalization. When including all patients, significant correlations were observed for CRP and ferritin, in such manner that higher CRP and higher ferritin levels correlated with lower sACE2 concentrations (Figure 5). Thus, the inflammatory state in severe COVID-19 may be associated with lower sACE2 levels. When stratifying for sex, we found no significant sex differences in how CRP and lymphocyte count were correlated with sACE2 (interaction terms: *p* = 0.26, *p* = 0.46). However, there was a statistically significant sex difference in how ferritin concentration correlated with sACE2 level (interaction term: *p* = 0.02) (Supplementary Figure 6).

## Discussion

The main finding of the present study is that sACE2 concentration decreases with the severity of COVID-19 among men, while sACE2 concentration increases with disease severity among women. We also found that the inflammatory biomarkers CRP and ferritin correlated inversely with sACE2 concentration in men, suggesting a role in severe disease. Our results indicate that sACE2 has potential to be a valuable biomarker of disease severity in patients with SARS-CoV-2 infection. However, sex has to be considered based on the different trends in sACE2.

Due to its role as the primary host cell receptor of SARS-CoV-2, several studies have speculated whether sACE2 levels could explain why some people are prone to develop severe disease. sACE2 may reflect both the level of membrane-bound ACE2 but also ADAM17 activity. As we did not analyze membrane-bound ACE2 and ADAM17, we were not able to decide to what extent they affected sACE2 levels in the present study. On one hand, elevated levels of sACE2 have been suggested to competitively inhibit the binding of SARS-CoV-2 to the membrane-bound ACE2, thereby protecting from disease progression (27). On the other hand, Swärd et al. proposed that high levels of sACE2 indicate increased ACE2 expression and elevated ADAM17 activity, leading to higher susceptibility to SARS-CoV-2 (28). In the present study, we found that sACE2 concentrations in men decreased with the severity of COVID-19. Our results are supported by another study where COVID-19 patients who were admitted to the ICU had lower sACE2 values than patients admitted to the ward or who were discharged (29). These findings are in line with the hypothesis that sACE2 plays a protective role in patients infected with SARS-CoV-2. Apart from competitively inhibiting binding of the virus, sACE2 may also protect from severe disease by reducing the activation of the renin-angiotensin system through negative feedback (30). Interestingly, we found an opposite trend in women where sACE2 concentration was increasing with disease severity. To our knowledge, we are the first to report these sex-dependent diverging trends in sACE2 concentration, which may be of particular interest as men are affected more severely by COVID-19 than women.

Our finding that healthy men have higher sACE2 than healthy women confirms previous results where higher sACE2 levels have been found in men from the age of 15 compared to women (28). Among patients with moderate COVID-19, we found the same pattern with higher sACE2 levels in men than in women. This is supported by another study including 114 hospitalized COVID-19 patients of which 22% were treated at an intermediate or intensive care unit (22). The sex difference disappeared when we analyzed sACE2 levels in severely ill patients requiring intensive care, illustrating the opposite trends where sACE2 concentration decreased with disease severity in men, but increased with disease severity in women. Since circulating sACE2 is sex-hormone dependent (31), the levels of sex hormones may play a partial role in these sex-related differences. More research is needed to confirm this and explain potential mechanisms. In the longitudinal analysis of sACE2 at hospital admission and at follow-up, we found no change in sACE2 levels. In line with this, Patel et al. (15) showed that the levels persisted at least 114 days post-infection. Thus, potential alterations in sACE2 after COVID-19 may require a longer time period than the present study spans.

Several risk factors for severe COVID-19, such as male sex, older age, hypertension, diabetes mellitus, high BMI, and heart failure are associated with chronically elevated levels of sACE2 (8, 32, 33). However, we found no difference in sACE2 levels in patients with cardiovascular comorbidities or with RAAS-blockade treatment. The latter may support the previous findings that RAAS-blockade is not associated with disease severity (19).

In the current study, the levels of CRP and ferritin inversely correlated with sACE2 concentration, although the correlation was rather weak. An increase in CRP and a decrease in sACE2 with disease severity were previously shown in a study from Spain with 963 patients tested for SARS-CoV-2 (29). Thus, the inflammatory state in severe COVID-19 may be associated with lower sACE2 levels. When stratifying for sex, however, the correlation between sACE2 and ferritin concentration was only seen for men, suggesting a sex-specific divergent trend. On the other hand, sACE2 was negatively correlated to CRP in female ICU patients, similar to men, despite the trend of increasing sACE2 with COVID-19 severity in women. Possibly, the mechanism(s) causing increased sACE2 with disease severity among women is overturned by the severe inflammatory state often seen in ICU patients. Alternatively, CRP may not be perfectly correlated to disease severity in critically ill women.

At present, there are no recognized models for predicting the disease course of COVID-19. Soluble ACE2 is involved in the pathophysiology of COVID-19, but its role as a biomarker of disease severity has been unclear. Our findings indicate that sACE2 has potential to be a valuable marker of disease severity in patients with SARS-CoV-2 infection. However, sex has to be considered based on the different trends in sACE2.

Limitations of this study include the single-center design and the relatively small number of individuals in the groups with healthy controls and patients with moderate COVID-19, especially women. The timing of blood sampling at hospital admission and follow-up differed between patients, which is also a limitation and could have affected the sACE2 levels. Moreover, lack of data regarding smoking habits is a limitation, since there is a hypothesis that ACE2 expression is upregulated in smokers which could increase their sensitivity to infection (34). Strengths of the present study include the comparison of sACE2 levels between three groups including healthy controls, hospitalized patients with moderate COVID-19 and patients with COVID-19 in the ICU. We have also performed a longitudinal follow-up of individual changes in sACE2 levels, which is not often seen in earlier studies.

## Conclusion

The decrease in sACE2 concentration, selectively in men, in severe COVID-19 is of pathophysiological interest since men are affected more severely by the disease compared to women. Additionally, the inflammatory biomarkers CRP and ferritin correlated inversely with sACE2 concentration in men, suggesting a role in severe disease. Our findings imply that sACE2 is a possible biomarker of disease severity in a sex-specific manner.

## Data availability statement

The raw data supporting the conclusions of this article will be made available by the authors, without undue reservation.

## Ethics statement

The studies involving human participants were reviewed and approved by the Swedish Ethical Review Authority (Dnr: 2020-01771). The patients/participants provided their written informed consent to participate in this study.

## Author contributions

JR, MG, and JS were responsible for the conception and design of the study, as well as for acquisition and analysis of data. BN collected the patients from the ICU. JR and SN performed the statistical analyses. LH and HZ performed the laboratory analyses. All authors took part in drafting the manuscript and approved the final version.
