# Supplementary material for: Sex difference in circulating soluble form of ACE2 protein in moderate and severe COVID-19 and healthy controls
Source: Front Med (Lausanne). 2022 Dec 8;9:1058120. doi: 10.3389/fmed.2022.1058120 (PMC9773379; doi:10.3389/fmed.2022.1058120)
Supplement: Supplementary file 1 [file Data_Sheet_1.PDF]

Supplementary material

This appendix has been provided by the authors to give readers additional information about their work.

Supplement to:

Sex difference in circulating sACE2 protein in moderate and severe COVID-19 and healthy controls

Josefina Robertson, Bengt Nellgård, Lillemor Mattsson Hulten, Staffan Nilsson, Keti Dalla, Mats Börjesson, Henrik Zetterberg, Joar Svanvik, Magnus Gisslén

Table of contents

Supplementary figures

Figure S1. Correlation between sACE2 concentration at hospital admission and age. 3

Figure S2. Correlation between sACE2 concentration at hospital admission and days since symptom onset. 4

Figure S3. sACE2 concentrations at hospital admission and follow-up in COVID-19 patients with and without corticosteroid treatment. Individual values and mean with SEM are shown for each group. Student's t-test was used for group comparisons. 5

Figure S4. sACE2 concentrations at hospital admission in COVID-19 patients with and without preexisting treatment with angiotensin-converting enzyme inhibitors (ACEi) or angiotensin receptor blockers (ARB). Individual values and mean with SEM are shown for each group. Student's t-test was used for group comparisons. 6

Figure S5. sACE2 concentrations at hospital admission in COVID-19 patients with and without cardiovascular comorbidities. Chronic heart disease includes coronary heart disease, heart failure, and cardiomyopathy. Individual values and mean with SEM are shown for each group. Student's t-test was used for group comparisons. 7

Figure S6. Correlations between sACE2 concentration at hospital admission and maximum level of C-reactive protein (CRP), maximum concentration of ferritin, and minimum lymphocyte count, stratified by sex, for patients with COVID-19 in the intensive care unit (ICU). 8

Supplementary figures

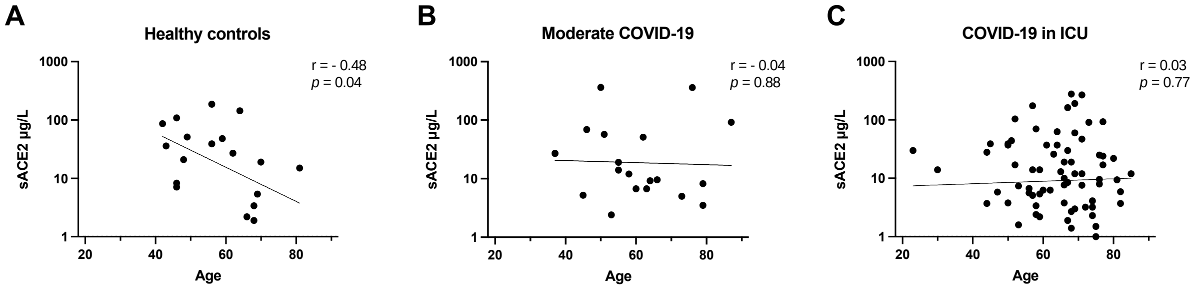

Figure S1. Correlation between sACE2 concentration at hospital admission and age.

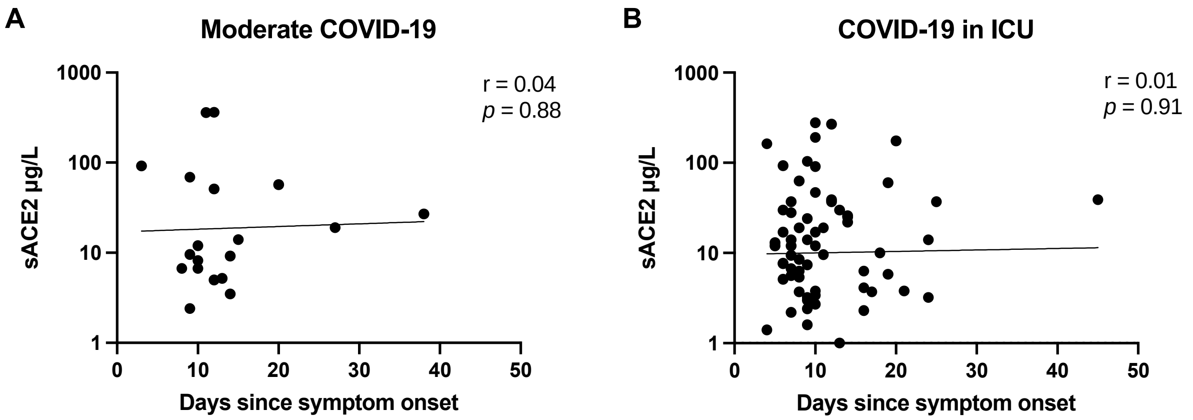

Figure S2. Correlation between sACE2 concentration at hospital admission and days since symptom onset.

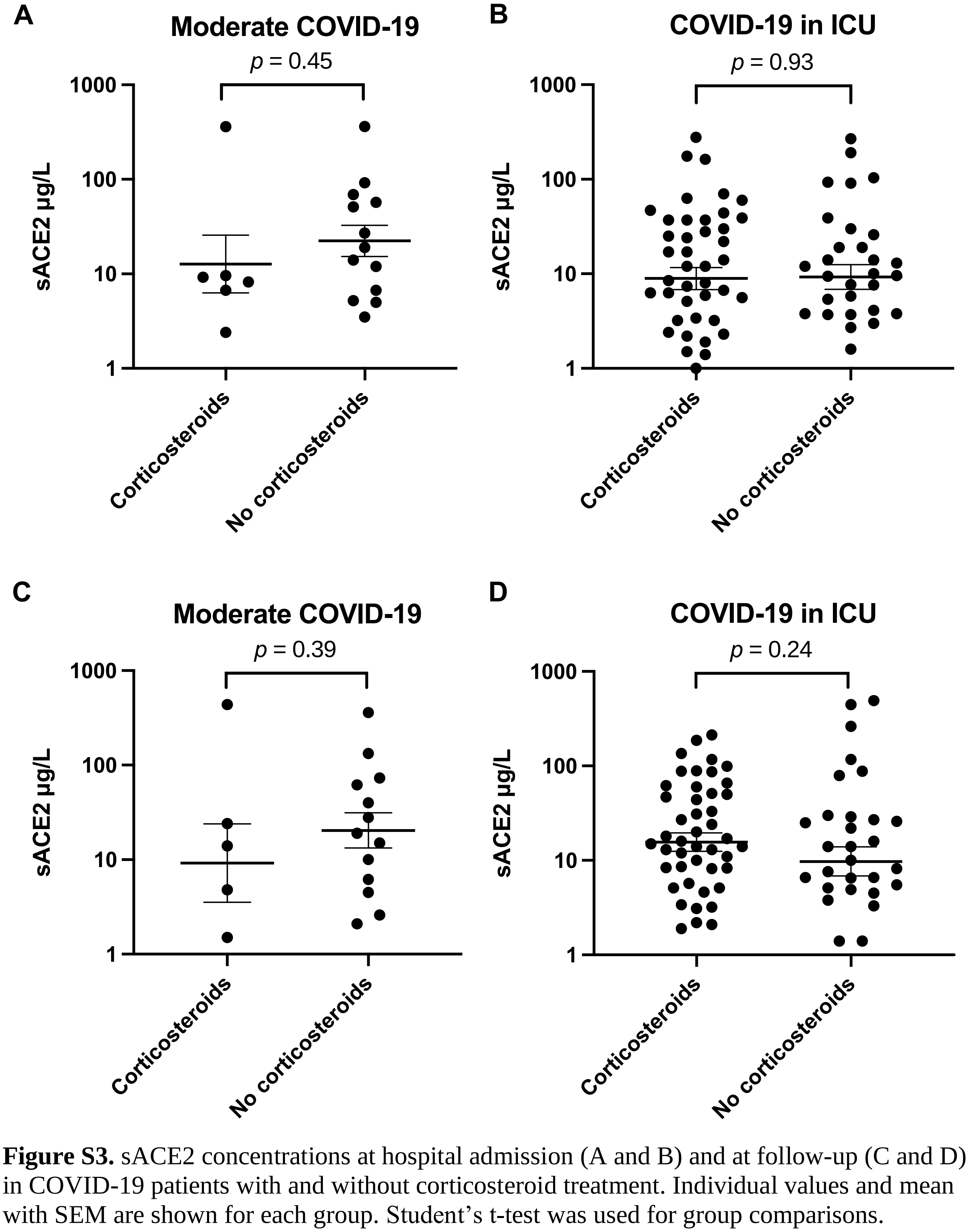

Figure S3. sACE2 concentrations at hospital admission (A and B) and at follow-up (C and D) in COVID-19 patients with and without corticosteroid treatment. Individual values and mean with SEM are shown for each group. Student's t-test was used for group comparisons.

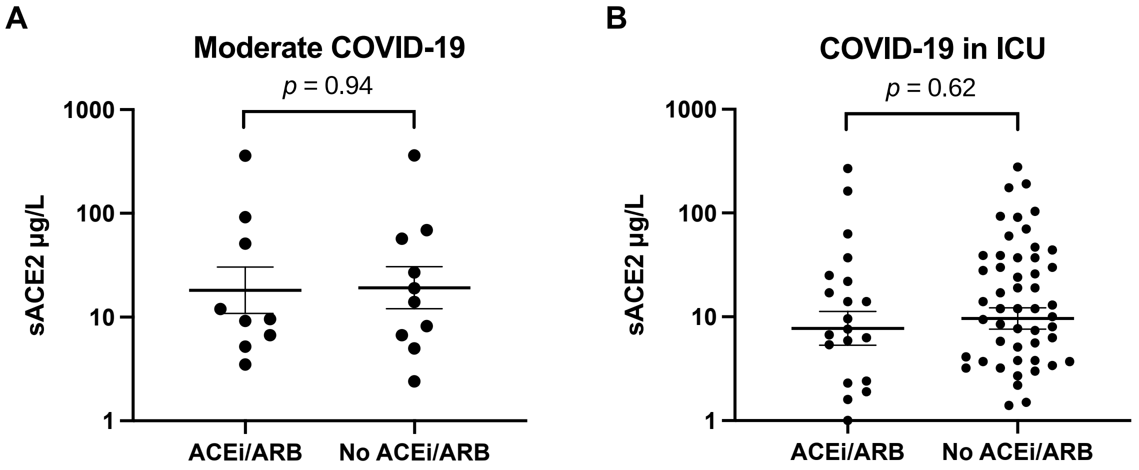

Figure S4. sACE2 concentrations at hospital admission in COVID-19 patients with and without preexisting treatment with angiotensin-converting enzyme inhibitors (ACEi) or angiotensin receptor blockers (ARB). Individual values and mean with SEM are shown for each group. Student's t-test was used for group comparisons.

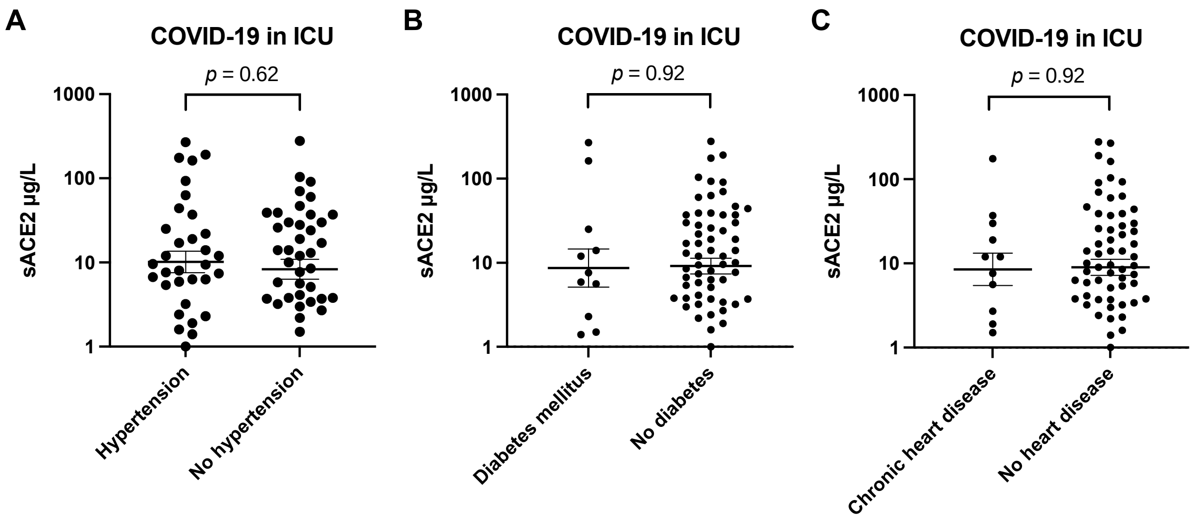

Figure S5. sACE2 concentrations at hospital admission in COVID-19 patients with and without cardiovascular comorbidities. Chronic heart disease includes coronary heart disease, heart failure, and cardiomyopathy. Individual values and mean with SEM are shown for each group. Student's t-test was used for group comparisons.

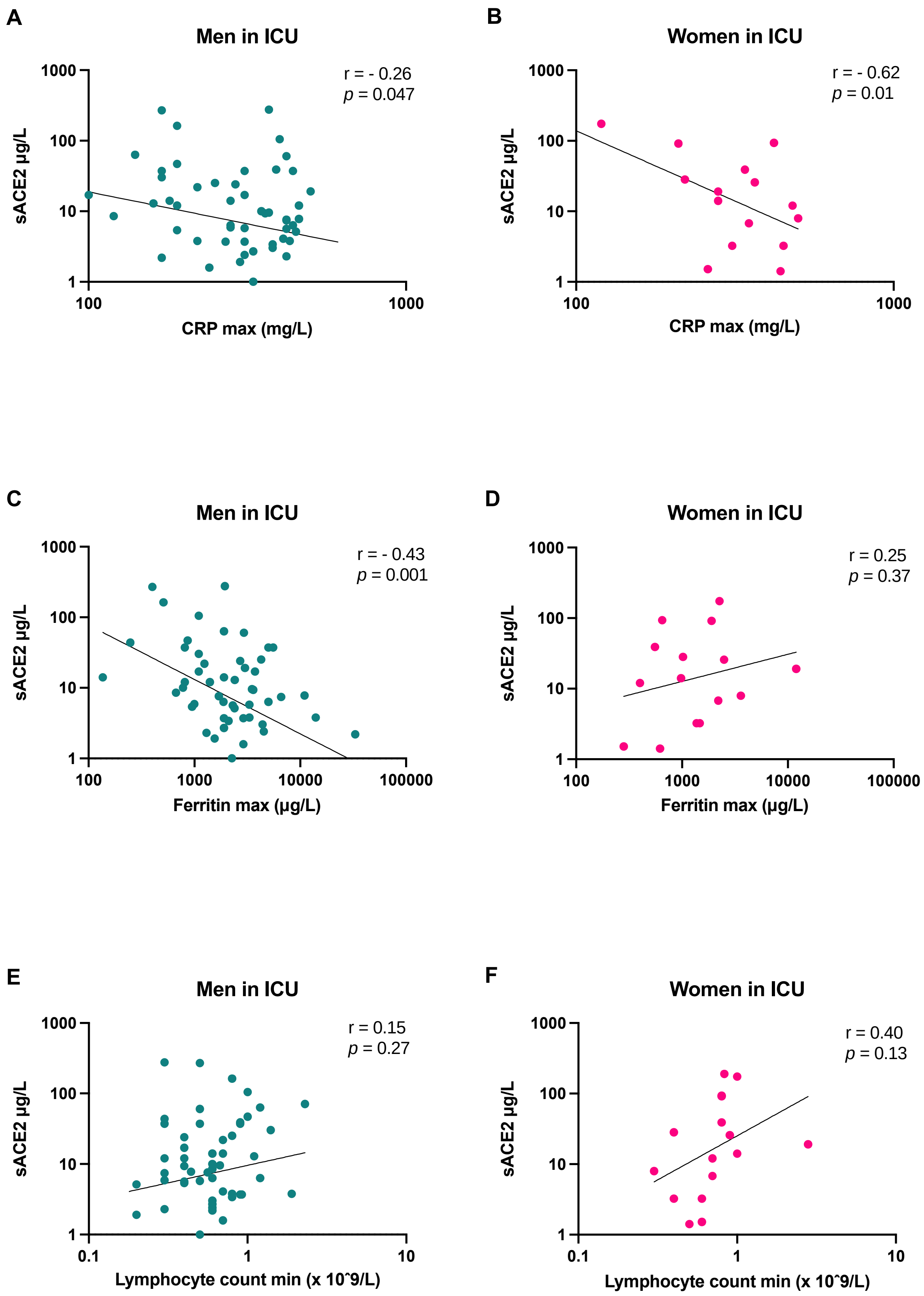

Figure S6. Correlations between sACE2 concentration at hospital admission and maximum level of C-reactive protein (CRP), maximum concentration of ferritin, and minimum lymphocyte count, stratified by sex, for patients with COVID-19 in the intensive care unit (ICU).
